# Navigating artificial intelligence in qualitative research: ethical and practical considerations from concept to publication

**DOI:** 10.3389/frma.2026.1863790

**Published:** 2026-06-18

**Authors:** Tyra Girdwood, Lydia Manikonda

**Affiliations:** 1 The University of Tennessee Health Science Center College of Nursing, Memphis, TN, United States; 2 Rensselaer Polytechnic Institute Lally School of Management and Technology, Troy, NY, United States

**Keywords:** artificial intelligence, critical reflections, ethical considerations, methodological challenges, mitigation strategies, qualitative analysis, qualitative research

## Abstract

The qualitative research field is evolving as artificial intelligence (AI) and its applications gain broader attention. While these new technologies may be of help to some aspects of the qualitative research process, they also pose several challenges that must be reflected upon, addressed, and documented. Some of the techniques offered by AI for qualitative analysis have existed for a long time, but hallucinated outputs and the more nuanced predictive abilities that may violate privacy and ethical aspects are of major concern. As this is an emerging field, there is a need for a synthesis of recommendations on AI use for qualitative work to reduce risks to rigor and reproducibility. In this paper, we identify key ethical and practical concerns and provide recommendations across five key stages of the qualitative research process so researchers and stakeholders can safely leverage AI to complement their human expertise.

## Introduction

The rapid rise of artificial intelligence (AI) technologies and advancements in Large Language Models (LLMs) have captured the attention of researchers and methodologists across several disciplines (Christou, 2023). In the qualitative field, novel tools like ©NVivo15's AI Assistant (2026) promise an accelerated way to summarize data, automate tasks, reveal patterns, and suggest analysis strategies, thus offering researchers a way to streamline their work. Notably, for researchers with access to large qualitative datasets that may be difficult to analyze, AI tools offer a new, potentially viable solution for the scalability and analysis of this data (Kuckartz and Rädiker, 2024). In fields like Natural Language Processing (NLP), work has been done to advance AI algorithms to perform highly complex qualitative analyses on large datasets like online forums (Fischer and Biemann, 2024; Williams, 2024). Additionally, in fields like medicine, nursing, and public health, patient data is protected by strong HIPAA rules which has led to the adoption of specially trained AI models to perform HIPAA-compliant qualitative work (Dankwa-Mullan, 2024).

Despite these advancements and the potential benefits, AI tools are raising critical epistemological, methodological, and ethical debates (Christou, 2023). While AI could enhance researcher productivity by streamlining the analysis process via AI-assisted coding, much of what AI currently produces lacks the vitality, depth, and authentic expertise of human intelligence (Jeon et al., 2025; Marshall and Naff, 2024). This is concerning as qualitative analysis techniques like thematic and narrative analysis focus on prioritizing depth and contextual richness over scale, so improper use of AI tools can create distrust in the rigor and reproducibility of qualitative work (Paulus et al., 2025). There is an additional need for qualitative researchers to co-develop human-in-the-loop AI frameworks with informaticists to improve AI tool performance while keeping qualitative ethical principles like informed consent, confidentiality, avoiding harm, transparency, respect for diversity, equity and power balance, and reflexivity at the forefront (Girdwood et al., 2026).

Although some preliminary cautions and recommendations exist, they tend to focus narrowly on one stage of the qualitative research process, usually the data analysis phase. For example, Williams (2024) described ethical challenges using NLP in the data analysis process, and Barrera et al. (2025) described how they used a human-in-the-loop process to oversee AI-facilitated transcription, coding, and thematic analysis. However, there is still no comprehensive synthesis of critical reflections on AI usage spanning all five stages of the qualitative research process. There is a need for more guidance on AI use for researchers to consider as they conceptualize ideas and designs, collect, analyze, and visualize data, and publish their qualitative work (Kuckartz and Rädiker, 2024; van Manen, 2023). Thus, we present key considerations and recommendations as researchers move across the lifespan of a qualitative project from concept to publication. Our goal is to discuss ways we can leverage AI tools and techniques carefully to support researchers, reduce harm, and enhance methodological rigor.

## AI usage in the qualitative research process: a synthesis of considerations

### Stage 1: concept formation and design

When formulating qualitative research questions, there are several nuances involved in creating innovative and/or significant questions. During this process, brainstorming for ideas and verifying the novelty and importance of such ideas via gaps in the literature can be a complex and tedious process. Bringing AI into this stage of the qualitative process may be beneficial as it can help search the literature quicker than a researcher who may have limited time and capacity (Phan and Le, 2025). However, not all references and ideas generated by AI may be relevant, and some references listed by the AI may be hallucinations (i.e., fake papers) which can significantly reduce rigor of the work before it even begins (Christou, 2023; Oermann et al., 2025). For example, while generating literature reviews on mental health, Linardon et al. (2025) found that approximately 66% of the citations were hallucinated or inaccurate, and scholars like Resnik and Hosseini (2026) argue the utilization of hallucinated citations could be considered research misconduct. Moreover, designs like Sandelowski (2000) qualitative description design have established methodological guidelines and approaches (like naturalistic inquiry) that conflict with AI-enabled data processes and analysis.

#### Thoughtful appraisal

We suggest asking these questions early on when considering using AI in your qualitative work: (1) Why is AI needed in my study?; (2) What work has already been conducted in this area and how does the use of AI fill gaps in the literature or knowledge?; (3) How does the use of AI align with my chosen qualitative research approach?; and (4) How do I keep human insights at the forefront of this work?

### Stage 2: recruitment and data collection

Recruitment of participants in qualitative studies can be difficult, especially if researchers are asking sensitive questions or looking for in-depth responses from harder to reach populations like children or marginalized individuals. A potential solution could be to leverage AI to generate synthetic participants in various forms through AI prompting, or giving a survey and asking AI to answer the survey with diverse personas (Gibson and Beattie, 2024). This may work best when generating simple datasets, however, when it comes to the diversity and richness of the data, AI cannot guarantee that it will generate high-quality information similar to that collected from human subjects (Gibson and Beattie, 2024). Relying on these AI systems is not advisable as they are trained on limited data and they possess limited capabilities in reasoning.

If data is collected through the recruitment of real human subjects, we could leverage AI to help conduct interviews, refine survey or interview questions, or to perform data transcription (Arbelaez Ossa et al., 2024; Jeon et al., 2025; Marshall and Naff, 2024). However, when using AI to collect interview data, the richness, notetaking, reflexivity, and depth of information may be diminished or lacking as compared to the data that an expert human interviewer would collect (Paulus et al., 2025). Additionally, there is a current lack of governance regarding accountability if AI gets it wrong, and since AI platforms are available to anyone in the world, data uploaded to them for tasks like data transcription must be fully de-identified to protect the confidentiality of participants (Arbelaez Ossa et al., 2024; Jeon et al., 2025; Marshall and Naff, 2024).

#### Thoughtful appraisal

At this stage, we suggest asking: (1) Can synthetic data or personas provide rich information to answer our research question(s)?; (2) How do human participants expect their data (audio and written) to be used and protected?; (3) How is the use of AI described in our consent forms?; and (4) Is it ethical to enter participant responses into an AI system that will use that information for training and AI-building?

### Stage 3: data analysis

Qualitative researchers understand that qualitative work is nuanced, iterative, and requires multiple individuals and their perspectives to interact with the same data to gain the most thorough inferences and insights (Paulus et al., 2025). Qualitative research isn't prescriptive like quantitative procedures that relatively stay the same, it requires the use of conceptual frameworks or the inductive generation of new themes and theories and back and forth discussions that enhance the richness of the data. We should be concerned that AI tools and programs could undermine or miss systemic barriers, social drivers of health, and social underpinnings that impact health and wellbeing, potentially leading to biases and harm to marginalized populations (Paulus et al., 2025). Some researchers have described AI systems as disproportionately representing individuals who identify as White and middle-to-high socioeconomic status, thus limiting diverse experiences and perspectives in AI algorithms and output (Capraro et al., 2024; Oermann et al., 2025).

Even though underlying LLM architectures are well studied and are currently used to advance AI capabilities, much of the generated content is difficult to interpret, unlike classical machine learning models. Due to the blackbox nature of these models, caution is required when using AI content for explainability purposes as it's unclear how AI insights are generated (Hitch, 2024; Williams, 2024; Zhang et al., 2025). Liao and Vaughan (2024) suggest four ways researchers could enhance transparency of LLM insights, including: reporting model documentation frameworks, publishing evaluation results on performance quality, fairness, and robustness, providing explanations of the LLMs overall logic and reasoning, and discussing uncertainty and limitations of LLM generated insights. Member checking (i.e., when participants review findings to ensure that the researcher's interpretations accurately reflect their experiences) is utilized in qualitative research to improve rigor and could also be adapted and used to assess the credibility and accuracy of LLM model interpretations.

Ethical and legal elements (i.e., HIPAA) associated with each dataset should be of prime concern when using AI for data analysis. For example, there have been several algorithms in the NLP field that have been developed to address various aspects of qualitative discourse, narrative, and thematic analyses (Williams, 2024). However, many of these algorithms were developed to analyze self-reported data that do not fall under strict HIPAA guidelines. Additionally, we must consider the possibility of data leakage issues among the AI systems that we have uploaded data to for analysis purposes. These data leaks can jeopardize participant privacy and confidentiality and open doors for emotional trauma and litigation. Once the data has been processed due to proprietary reasons, users of these systems are not aware of how their data is being used, stored, or analyzed.

#### Thoughtful appraisal

During the data analysis stage, we recommend investigating whether there is: (1) Inaccurate, biased, or fabricated interpretations created by AI; (2) Lack of transparency or verifiability in AI outputs; (3) Poor consistency, accuracy, or data protection in AI performance; (4) User difficulty in designing AI prompts to analyze data; (5) Costs associated with AI programs like ChatGPT to analyze data; and (6) Confidence in how the AI understands the qualitative data and the best ways to analyze it.

### Stage 4: data visualization

A big draw researchers have to AI systems is their versatility in visualizing data. Using AI programs like ©Canva can be helpful for researchers who have trouble creating visual diagrams or figures, as it is rather easy for end users to write a prompt and send the data for the system to generate visually appealing images (Phan and Le, 2025). Researchers should note the importance of writing AI prompts correctly to generate content that is envisioned by the end user. Data visualization is successful if all the C's (i.e., Clear, Concise, Consistent, Correct, and Compelling) are addressed in the generated visual (The 5 C's of Effective Data Visualization, n.d.).

Another potentially useful AI feature for qualitative research is the ability to produce AI-replicas of original images to help keep real human participants anonymous. However, the original image must be inputted into the AI system to create such replicas, essentially giving away biometric data which can then be stored, trained on, or sold to other companies (Phan and Le, 2025). These AI-replicas are different from deepfakes, which are images that are solely created by AI to depict fabricated or fake events (Phan and Le, 2025). While there are some benefits to AI data visualization, there are also challenges that arise from a mismatch of mental models of the end user (communicated via prompts to the AI), and the AI system understanding and generating content appropriate to the given prompt. AI might not fully understand directions/prompts asking it to design a certain image or visualization, causing confusion for the qualitative researcher (Phan and Le, 2025). AI has limits in the amount of detail it can produce in images that may lead to communication fatigue on the end user. There are also concerns regarding data privacy where one may need to use general descriptions in the prompt making process to keep participants and their data anonymous (Phan and Le, 2025).

#### Thoughtful appraisal

At this stage, we suggest researchers consider: (1) If AI-generated images or graphics are necessary for the qualitative work; (2) If any AI-generated visualizations protect participant anonymity; and (3) Whether the visualizations accurately display quotes or inferences made from the qualitative findings.

### Stage 5: writing for publication

A recent poll of 5,000 researchers found contrasting views on when it is acceptable to use AI in the writing of manuscripts and what needs to be disclosed (Kwon, 2025). Most of the poll participants agreed that using AI to draft a manuscript's abstract is acceptable, but many disapproved of the use of AI to write key sections of a manuscript. As noted by Khalifa (2024), AI could be useful to enhance academic writing in six areas: idea generation, content structuring, literature synthesis, data management, editing, and ethical compliance. Many of the AI systems are demonstrating significant potential in areas like enhancing English translations for non-English speakers, but human-led oversight and manual checking are still needed to maintain academic integrity and trustworthiness in the qualitative community (Oermann et al., 2025). Notably, AI “tools, methods, and models are only as good as the data used to train them” (Oermann et al., 2025).

Moreover, when submitting a qualitative manuscript to a journal, it's critical for authors to add a strong AI disclosure statement that includes the AI tool's name and version used, specific tasks the AI performed (e.g., editing, coding, visualization), any boundaries placed on the AI tool (e.g., “*no content or interpretations were generated by AI*”), degree of human oversight (e.g., “*the authors reviewed for accuracy and take full responsibility of all AI-generated content*”), and ethical practices that were utilized (e.g., “*all data uploaded to AI were deidentified*”).

#### Thoughtful appraisal

During this final stage, we suggest: (1) Not including an AI tool as an author on your manuscript; (2) Reviewing references for potential hallucinations (i.e., fake references); (3) Running your manuscript through a plagiarism checker like ©Turnitin; (4) Disclosing purposes and rationale for using AI in your work; (5) Addressing limitations of AI usage in the discussion section of the manuscript; and (6) Following each journal's guidance and/or policies surrounding the use of AI in qualitative work.

## Mitigating artificial intelligence challenges

We acknowledge there are several challenges in leveraging AI for qualitative research. We propose mitigation strategies (see Table 1) and critical reflections (see Table 2) that researchers and stakeholders could utilize to enhance rigor, transparency, and reproducibility of AI usage in their qualitative work. While we offer these suggestions, it is important to note this is an emerging field that continues to be shaped by new recommendations. Individuals interested in using AI for qualitative research should stay abreast of updated guidance (i.e., follow qualitative AI workgroups/consortia and read recent publications), consult with trusted mentors and qualitative and AI experts, and continue to justify decisions made to use AI in their qualitative work to help shape this growing qualitative AI era.

**Table 1:** Multi-level mitigation strategies for AI challenges.

Level	Strategies to mitigate AI challenges
Researcher	•Researchers should transparently explain how and why AI was used in their qualitative work
	•Researchers can adapt established qualitative reporting checklists (e.g., COREQ and SRQR) to report AI usage (O'Brien BC, 2014; Tong, 2007)
	•Researchers across disciplines should begin to develop and test AI-specific frameworks and theories that could provide structure for AI use in qualitative work
Organization	•Academia: Faculty should update academic curricula to include ethical and practical considerations of using AI in qualitative research
	•Teams and organizations with interdisciplinary perspectives should co-design AI governance for qualitative work (i.e., individuals across health, technology, informatics, communication and other disciplines, and broadly across academia, government, industry, and various communities)
Qualitative community	•Qualitative research consortia/institutes should construct standards and policies for an AI methodology as a new digital approach in qualitative research

To move this field forward, these researcher-level, organization-level, and qualitative community-level strategies could help us begin to address AI challenges we are facing.

**Table 2:** Critical reflections on integrating ai across qualitative research stages.

Stage in the qualitative process	Ethical	Practical
	Considerations	Considerations
Stage 1: concept formation and design	Am I clear about the boundaries of what AI should and should not do?	Have I articulated *why* AI is needed/critical to this work? Have I described how AI aligns with my chosen qualitative design/approach?
	Am I aware that AI may generate irrelevant or misleading references?	Have I verified novelty and importance of my research question through a human-led literature review?
Stage 2: recruitment and data collection	Have I confirmed whether the AI tool complies with HIPAA or other privacy regulations? Have I communicated data protection measures transparently in my work and to participants?	*If considering synthetic data*: Am I aware that AI-generated people may lack diversity, nuance, and authenticity? Have I evaluated whether synthetic “participants” are appropriate for the research purpose?
	*If considering AI for transcription:* have I ensured no identifiable or sensitive data will be uploaded or stored by the AI system? Does the consent form clearly describe how audio/text data will be processed?	*If using AI to help collect data*: Have I tested how different prompt styles/language affect the quality of data collection?
Stage 3: data analysis	Have I checked for hallucinations, inaccuracies, or fabricated content created by the AI?	Can an AI-assistant or tool truly *understand* qualitative data in the way a human researcher does? Can AI meaningfully interpret context, nuance, and emotion?
	What biases or blind spots might AI introduce? Have I considered how AI's lack of interpretability affects trustworthiness?	Are the AI insights verifiable, transparent, and grounded in the data?
	Who is accountable if AI-generated insights are wrong?	Have I documented how AI was used in the analytic process? Have I documented procedures taken to enhance rigor and reproducibility of the findings?
Stage 4: data visualization	Am I using general descriptions to protect participant anonymity? Does the visualization risk revealing participant identity?	Have I written a clear, specific prompt describing the desired visualization? Have I checked whether the AI tool can handle the level of detail required?
	Have I confirmed that AI replicas are not deepfakes or misleading representations?	Does the AI-generated visualization accurately reflect the data?
Stage 5: writing for publication	Have I ensured AI is not listed as an author?	Have I verified all references and citations for accuracy and existence?
	Have I reviewed content for gender, racial, or cultural bias?	Have I disclosed how AI was used in the manuscript?
	Have I described limitations of AI use in the discussion?	Have I followed the journal's AI policy and guidelines?

We suggest researchers and stakeholders critically reflect on (and document) these considerations during each stage of the qualitative process when leveraging AI in their work.

## Data Availability

The original contributions presented in the study are included in the article/supplementary material, further inquiries can be directed to the corresponding author.
